# Autologous mesenchymal stem cell transplantation for spinal fusion: 10 years follow-up of a phase I/II clinical trial

**DOI:** 10.1186/s13287-023-03298-4

**Published:** 2023-04-11

**Authors:** Victoria Gomez-Ruiz, Juan F. Blanco, Eva M. Villarón, Helena Fidalgo, Miriam López-Parra, Fermín Sánchez-Guijo

**Affiliations:** 1 Department of Orthopedic Surgery, IBSAL-University Hospital of Salamanca, Salamanca, Spain; 2 Cell Therapy Area, Department of Hematology, IBSAL-University Hospital of Salamanca, Salamanca, Spain; 3 Department of Surgery, University of Salamanca, Salamanca, Spain; 4 Department of Medicine, University of Salamanca, Salamanca, Spain; 5 Network Center of Regenerative Medicine and Cellular Therapy of Castilla y León, Salamanca, Spain

**Keywords:** Mesenchymal stem cells, Spinal fusion, Bone graft, Intervertebral disc degeneration, Cell transplantation

## Abstract

Posterolateral spinal fusion is the standard surgical approach for patients with degenerative disc disease. In our previously published article, we reported a 5-years follow-up of a phase I/II clinical trial in patients undergoing spinal fusion with autologous mesenchymal stem cells (MSCs) embedded in tricalcium phosphate. In the current manuscript, we have updated the results with a 10-year follow-up, the longest reported to date in this setting. After clinical and radiological evaluation, safety of the procedure was further confirmed in all 11 treated patients, with no evidence of tumor, infection, inflammatory reaction, or heterotopic ossification related to the administration of MSCs. Regarding clinical efficacy, low back pain and radicular pain (both assessed by the visual analogue scale—VAS), and the Owestry Disability Index remained significantly lower compared to pre-intervention. Radiologic evaluation demonstrated spinal fusion in all cases, improving over time. Finally, quality of life improved significantly also during follow-up. In summary, the use of tricalcium phosphate-embedded autologous MSCs with lumbar posterolateral arthrodesis is safe and potentially provides long-term benefits for 10 years.

To the editor,

The gold-standard surgical approach to treat lumbar degenerative disc disease (DDD) is posterolateral spinal fusion [1]. Generally, the procedure is complemented by the addition of autologous or allogeneic bone grafts or bone substitutes. Although high fusion rates can be obtained with autologous bone graft, the procedure has some disadvantages, such as pain and increased surgical time [2].

The use of mesenchymal stem cells (MSCs) in spinal fusion procedures has raised as a potentially attractive approach, based on the ability of these cells to differentiate into osteoblast together with their strong anti-inflammatory and immunomodulatory capacity [3]. Although benefit has been suggested in animal models [4], information on its clinical application is scarce.

In a previous work [5], we presented the results of a phase I/II clinical trial of MSCs with tricalcium phosphate combined with spinal fusion for DDD. In that initial report, we provided the 5-year follow-up for the 11 patients treated. In the current manuscript, we provide the outcome after 10-years.

For the 10-year follow-up analysis, the 11 patients previously reported were assessed in person both clinically and radiologically. As in the previous study, pain was evaluated by means the visual analog scale (VAS), functional status by means of the Oswestry score and physical functioning, assessing this subsection of 10 items from the SF36 questionnaire. Lumbar spine X-ray was also performed. Updated data on comorbidities and complications were also collected.

Statistical analyses were performed in R 4.2.0. All numerical variables followed a normal distribution (Shapiro–Wilk test). For the comparative analysis of quantitative variables, the Student’s t-test for paired samples was used comparing pre-intervention versus 10 years, pre-intervention versus 1 year, and 1 year versus 10 years. In addition, an Anova test for paired samples was performed to compare the three experimental groups.

The most important finding of the long-term evaluation after 10 years is the fact that no late adverse events were observed in this period after in-person clinical and radiological evaluation of the treated patients. These includes local tumoral transformation, local infections, or lumbar heterotopic ossification related to the administration of MSCs. Solid radiological fusion was present at this late timepoint in 100% of the patients, without instrumentation loosening, segmental instabilities, lytic areas or pseudoarthrosis. The updated 10-years analyses showed a maintained significant decrease (*p* = 0.026) of low back pain intensity compared to the pre-surgery assessment (Fig. 1A). The VAS scale for radicular pain (Fig. 1B) was also significantly lower at this late timepoint (*p* = 0.046). Regarding disability evaluation, the Owestry index (Fig. 1C) was significantly decreased 10 years after surgery (*p* = 0.015). Finally, 10-years physical functioning evaluation from the SF-36 questionnaire (Fig. 1D), was also significantly improved compared to that of the pre-surgical status (*p* = 0.026).

**Fig. 1:**
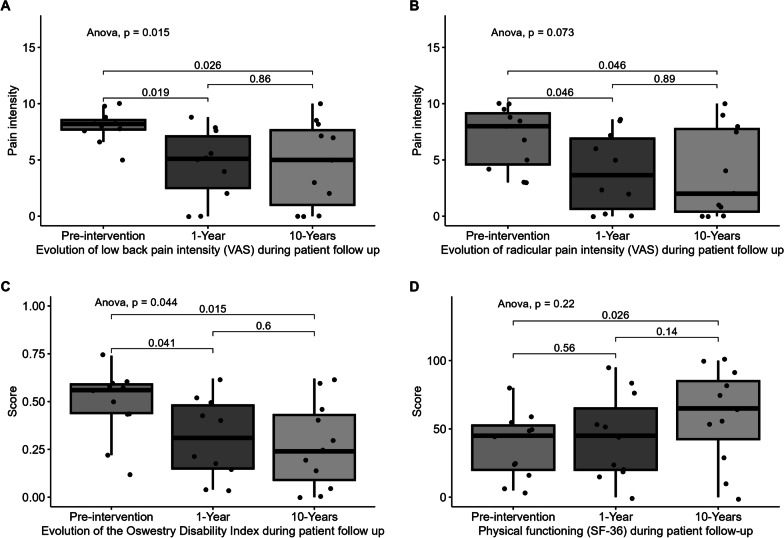
Evaluation of pain, disability and physical functioning after 10 years compared to the first year and to pre-intervention

In summary, our 10-year evaluation results show that the use of autologous MSCs embedded in a tricalcium phosphate scaffold in addition to posterolateral arthrodesis is a safe procedure and suggest potential benefits at the clinical, radiological, and physical functioning levels, that should be demonstrated in larger phase II or III randomized trials.

## Data Availability

The datasets generated and analyzed during the current study are included in this published article. Additional data analyzed during this study are available from the corresponding author on reasonable request.

## Abbreviations

MSCs: Mesenchymal stem cells
VAS: Visual analog scale
DDD: Degenerative disc disease
